# Many roads to minimizing regret: A comparison of Wang et al (2024) and OpAL* models of adaptive striatal dopamine

**DOI:** 10.1371/journal.pcbi.1012920

**Published:** 2025-05-08

**Authors:** Joshua T.S. Hewson, Alana Jaskir, Michael J. Frank

**Affiliations:** 1 Carney Institute for Brain Science, Brown University, Providence, Rhode Island, United States of America; 2 Department of Cognitive & Psychological Sciences, Brown University, Providence, Rhode Island, United States of America; 3 Department of Psychology, New York University, New York, New York, United States of America

Wang et al (2024) [1] introduce a powerful extension to their model of the basal ganglia, in which dopamine (DA) encoding of novelty facilitates efficient uncertainty-driven exploration. The model builds on previous formulations in which direct and indirect (D1 and D2) striatal pathways represent the mean and variance of reward distributions [2]. The new model suggests that dopaminergic novelty signals have an adaptive effect, dynamically modulating the contributions of these pathways to resolve an exploration-exploitation tradeoff as a function of uncertainty. The model is shown to account for electrophysiological recordings and behavioral data. Moreover, the authors showed that this scheme is normative in that, when its parameters are optimized for different task environments, it outperforms several baseline models.

The authors’ model is clever and generates novel testable predictions. Here we offer some commentary on their comparison to a related neurally-inspired model of the basal ganglia, OpAL* (pronounced *opal*, as in the gem stone; [3]). Their simulations suggested that OpAL* fails at certain task contingencies, challenging Jaskir & Frank’s (2023) [3] claims regarding its normative advantages across tasks with varying reward contingencies and number of choice alternatives. Wang et al (2024) [1] moreover highlighted the lack of applicability of OpAL* to tasks with continuously distributed (Gaussian) rewards. Upon further inspection, however, we find that these conclusions are largely artifactual and that OpAL* compares favorably in all task contingencies. We elaborate these points with simulations below. We suggest that differential predictions of these models should be compared empirically and speculate how they may be fruitfully combined.

In the original OpAL model [4], D1 and D2 pathways learn actor weights from positive and negative reward prediction errors (RPEs), but in opposite directions. Importantly, the learning rule has a nonlinear Hebbian “activity-dependent” component, such that the weight change is also dependent on the level of activity in the corresponding D1 or D2 population. This recursive update rule causes the two distinct D1 and D2 actor weights to specialize in discriminating between actions with high and low reward probabilities, respectively (for details, see [3]). Moreover, OpAL* introduced a mechanism to dynamically alter its DA levels as a function of environmental reward richness. As in empirical data (e.g., [5]), DA levels increase when the history of recent rewards is rich, and decrease when they are sparse. Jaskir & Frank (2023) [3] showed that this adaptive DA modulation can amplify the contributions of the neural pathways best suited for the task, akin to an efficient coding strategy (see also [6]) . These mechanisms make OpAL* outperform standard models such as Q-learning and Upper Confidence Bound (UCB), as it expediently optimizes its policy across varying reward environments [3].

In contrast, Wang et al (2024) [1] found that OpAL* performed starkly suboptimally in comparison to the other models in their settings (Fig 5A and 5B). On closer investigation, we noticed some errors in the OpAL* implementation and a misleading comparison with optimization procedures. When corrected, OpAL* shows favorable performance and both OpAL* and the model proposed in [1] exhibit excellent benchmark performance, suggesting they each have normative properties.

First, for all models except OpAL*, the authors used an optimizer to search for the parameters that optimized performance. In contrast, for OpAL*, performance was evaluated using a coarse grid search over a restricted range of parameters, resulting in unequal model comparison. [This grid was used in the 2023 OpAL* article [3], but for a different purpose: to demonstrate that adaptive DA aids performance across a wide range of model parameters (e.g., learning rates and softmax temperatures) for exploration, were suboptimal. Biologically there are many factors that can lead to variations in these parameters, such as and norepinephrine levels [7–9])]. Specifically, in [1], the softmax beta parameter (controlling exploration) was restricted to be between 1 and 10, but the task environments simulated included a larger action space with 10 alternative actions – a scenario that requires a great degree of exploration. By limiting the lower bound of beta to 1, the model could not effectively explore across actions. We confirmed this intuition using Wang et al’s (2024) [1] code base. For example, in their bandit problem with 0.9 vs 0.8 reward probabilities and 10 arms, OpAL* showed quite reasonable performance when beta parameters were lowered below 1 (e.g., for a simple parameter set of [0.25, 0.25, 0.25]).

**Fig 1:**
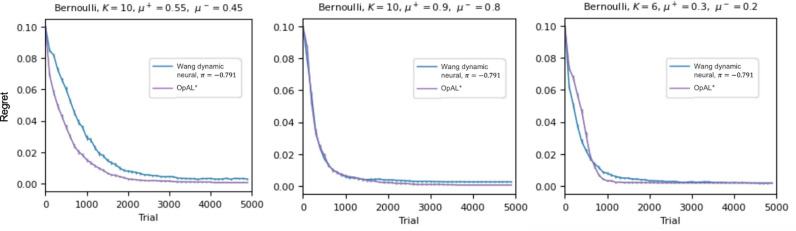
Mean regrets across 1000 simulations for Wang et al’s [1] best neural model across all their simulations (“dynamic”, π = -.791) in blue; OpAL* in purple, using the same Bernoulli conditions considered in [1], Fig 5. Lower is better. Other comparison models are omitted here for clarity of comparison, but these are all worse and can be seen in [1] Fig 5. OpAL* parameters used here are [.01, .01, 0.5], [0.44, 0.19, 0.44], and [0.02, 0.5, 0.13], respectively.

Aside from this difference in optimization methodology, we also noticed other minor errors and discrepancies in the authors’ OpAL* implementation: (i) G/N weights were initialized to 0.5. OpAL* initializes them to 1 due to the multiplicative nature of the Hebbian term, such that the impact of the first RPE reduces to a standard actor update rule; ii), actor weights were capped at 10 for an unknown reason; iii) The *T* parameter, which controls annealing of actor learning rates, was set to 10, whereas Jaskir & Frank (2023) used 100. We corrected these discrepancies (adapted code can be found here: https://github.com/TheLemonPig/CompareBGOpal). We then conducted our own optimization procedure, in which we optimized three parameters: the critic learning rate, the actor learning rates (constrained to be equal across actors), and the softmax temperature. Because other parameters could in principle also be optimized, including asymmetric actor learning rates and dynamic DA modulation parameters *k* and *phi* (which were fixed to the same values used in [1] and [3] for consistency), results can be treated as a lower bound for performance. We used the OpAL* implementation code provided by [1] (with our corrections) and the same *shgo* optimizer used by [1] for their models. The optimizer sometimes produced inconsistent parameter results, so it cannot be guaranteed that our results represent the global optimum. Nonetheless, simulations from these optimized values yielded excellent OpAL* performance in the three Bernoulli settings for which Wang et al’s [1] best neural model outperformed all other models (Fig 1). [Note that Wang et al. also considered other variants of their model but they generally performed worse except for one case (first panel, Fig 1); OpAL* still performed favorably relative to their optimal model in that scenario].

**Fig 2:**
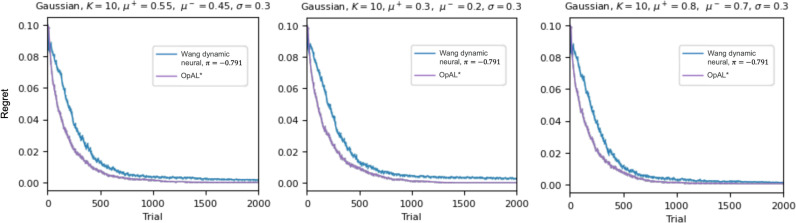
Gaussian outcomes. Mean regrets across 1000 simulations for Wang et al’s [1] best neural model across all their simulations (“dynamic”, π = −.791) in blue; OpAL* in purple. Left, Gaussian outcomes considered by [1], for which both models were optimized. Middle and right, applying these optimized parameters to task contingencies with lower and higher mean reward statistics. X-axis limited to 2000 trials to zoom in on learning differences; performance was found to be stabilized by then for both models. OpAL* parameters are [.01, 0.38,.25].

In Gaussian continuous reward environments, Wang et al [1] omitted OpAL* as a comparison, suggesting that it was not defined in this case. But while Jaskir & Frank (2023) [3] focused on binomial settings, it is simple to apply OpAL to the Gaussian setting considered in [1], where outcome distributions overlap for the best and worst options. Here, OpAL actors can still learn from continuous RPEs to prefer those actions that consistently yield the best outcomes on average. Indeed, we found that OpAL* performance exceeded that of the other models for the contingencies considered by [1] (Fig 2 left). To explore whether this conclusion held for other Gaussian distributions with varying means, we simulated performance for both models (using the same parameters that had been optimized for the above setting). Excellent OpAL* performance was still observed for higher and lower mean reward values (Fig 2) [Note though that a condition for good performance is that critic values should be initialized within the range of the reward statistics for these options (e.g., V(0) = 0.75 for mu = 0.8 vs 0.7), allowing G/N weights to favor actions that produce consistently RPEs that are largely consistent in sign over the course of learning. For fair comparison, this initialization was also provided to Wang et al’s neural model initial Q values, which also helped its performance. Moreover, a nearly equivalent implementation would be to allow the agent to sample a few actions and initialize allow the critic to the mean outcome; alternatively, the agent could simply be forced to select each option once before using its policy (like UCB).].

How does OpAL* succeed in these Gaussian settings despite substantial overlap in distribution between options? As described by Jaskir & Frank (2023) [3], its opponent actors show enhanced sensitivity to the consistency (in sign) of the history of positive and negative RPEs, respectively, for each action. For Gaussian outcomes, the G actors emphasize differences in the upper tails of the outcome distributions, while N actors emphasize differences in the lower tails (again due to efficient coding). In combination, these actors can expediently optimize performance. We confirmed that OpAL* performance was facilitated by its opponent actors; indeed, using only the G actor for example largely eliminated the OpAL* advantage.

Notably, in these Gaussian cases we found that OpAL performance was also excellent when removing the meta-critic and thus disabling dynamic DA modulation (i.e., k = 0). Nevertheless, we anticipate that performance could be further optimized with a more sophisticated meta-critic for distributional settings. Here we simply emphasize that the nonlinear actor weights in OpAL can enhance discrimination between Gaussian outcomes with overlapping distributions. [In the simulations reported above we did use the OpAL* metacritic with k = 20, for consistency with [3]. This meta-critic used a Beta distribution to track reward probability. So as to not introduce new changes for this commentary, above we used that same meta-critic, but where the Beta distribution tracked probability of positive RPEs instead of raw rewards (these are equivalent in the binomial case since each reward is a positive RPE; hence the metacritic remained unchanged from [3]). But there is no advantage of DA modulation in this setting, and hence performance was unaffected by removing the meta-critic altogether. We anticipate that this scheme may still be useful in hybrid settings with probabilistic rewards and variable magnitudes, which is only a slight modification from the adaptive risk-tasking simulations in [3]].

Overall, both neurally inspired models (OpAL* [3] and Wang et al, 2024 [1]) performed quite favorably even relative to other algorithms explicitly designed to optimize exploration vs exploitation. Thus, future studies should consider testable predictions that may arbitrate between them empirically. While space limitations preclude a full discussion here, please see Jaskir & Frank (2023) [3], Collins & Frank (2014) [4], and Frank (2025) [6] for reviews of empirical phenomena consistent with nonlinear Hebbian plasticity in the OpAL* model that could not be easily accounted for otherwise. Undoubtedly, however, there are also patterns of data in which novelty DA responses may be useful for explaining performance. Thus, it may be fruitful to combine these objectives, for example in a meta-critic that considers both novelty and environmental reward richness, and to evaluate how these might interact with the different sorts of nonlinearities assumed in the actors of both models. Such a combined approach could potentially leverage the advantages of both models to explain a wider range of data.
